# Low Temperature Suppress Cell Wall Degradation by Modulating Antioxidant Metabolism in Figs

**DOI:** 10.3390/foods15142418

**Published:** 2026-07-08

**Authors:** Lingci Ge, Di Zhao, Ping Wang, Bing Xie, Zuoli Zhang, Xiangchao Shangguan

**Affiliations:** College of Food Science and Engineering, Tarim University, Aral 843300, China; gelingci@163.com (L.G.); zzdd0102@163.com (D.Z.); wpaing513@163.com (P.W.); x912439359@163.com (B.X.); zlzhang202309@taru.edu.cn (Z.Z.)

**Keywords:** figs, storage temperature, postharvest quality, antioxidant metabolism, cell wall degradation

## Abstract

The effects of low (4 °C) versus ambient (25 °C) temperature on cell wall turnover, and redox homeostasis in ‘Jin’Aofen’ figs were investigated. Compared with 25 °C, storage at 4 °C reduced decay and respiration rates, delayed firmness loss and color deterioration. It also suppressed PG, PME and CEL activities, maintained higher contents of protopectin and cellulose; and slowed soluble pectin accumulation. Storage at 4 °C maintained higher activities of SOD, CAT, POD, APX and GR. It also preserved the levels of non-enzymatic antioxidants, including TPC, TFC, ASA GSH. Meanwhile, it reduced H_2_O_2_ and O_2_^−^ production and inhibited MDA. In addition, storage at 4 °C suppressed PPO, thereby alleviating enzymatic browning. Overall, storage at 4 °C maintained postharvest quality of figs by coordinately inhibiting cell wall degradation and regulating antioxidant metabolism.

## 1. Introduction

Fig (*Ficus carica* L. cv. ‘Jin’Aofen’) is a perennial deciduous fruit tree belonging to the genus *Ficus* of the Moraceae family. ‘Jin’Aofen’ is the dominant late-maturing fig cultivar cultivated on a large scale in the Xinjiang Production and Construction Corps. Its fruit is valued in the marketplace for its distinctive flavor and substantial nutritional profile [1], with high levels of calcium, potassium, phosphorus, vitamins B, C, K, and dietary fiber [2]. In addition, figs contain abundant bioactive compounds, such as phenolics, flavonoids, phenolic acids, and polysaccharides, which contribute to their strong antioxidant capacity and potential health-promoting properties [3]. However, harvested figs exhibit a vigorous postharvest respiration rate. Their soft texture, thin skin, high moisture content, and the specific structure of the basal ostiole predispose figs to mechanical injury and microbial spoilage during postharvest handling. These vulnerabilities lead to rapid softening, decay, and quality deterioration. Consequently, the shelf life of fresh figs under ambient conditions is limited to merely 2–3 days [4,5], which severely restricts its long-distance transportation and market circulation in southern Xinjiang.

Postharvest fruit deterioration is a complex process associated with cell wall disassembly, oxidative stress, and browning [6]. The plant cell wall is a key structural basis for maintaining fruit texture and firmness [7]. Its major components, pectin and cellulose, play important roles in cell adhesion, structural support, and protection [8,9]. During fruit ripening and senescence, cell wall degradation leads to tissue loosening and firmness loss. The enzymatic machinery responsible includes polygalacturonase (PG), pectin methylesterase (PME), and cellulase (CEL). These enzymes collectively modify the structural framework of the cell wall by hydrolyzing pectic substances and cellulosic fibrils, thereby accelerating fruit softening [10].

Reactive oxygen species (ROS) are a class of highly oxidizing oxygen-containing substances, including hydroxyl radicals (·OH), hydrogen peroxide (H_2_O_2_), singlet oxygen (^1^O_2_) and superoxide anion (O_2_^−^) [11]. Under normal physiological conditions, ROS metabolism in fruit and vegetable tissues maintains a dynamic balance. However, postharvest stress and senescence disrupt this balance, resulting in excessive ROS accumulation and oxidative stress. This process causes oxidative damage to the cell membrane system, proteins, and lipids, and accelerates the process of fruit quality deterioration and senescence [12,13]. Increasing evidence also indicates a close interaction between ROS metabolism and cell wall metabolism. Li et al. [14] reported that during peach softening, ·OH directly attacked cell wall polysaccharides, leading to depolymerization of soluble pectin and cellulose. ROS may also promote fruit softening by regulating the activities of cell wall-degrading enzymes [15].

Plants rely on a sophisticated antioxidant system of enzymatic and non-enzymatic parts. This system scavenges excess ROS and prevents oxidative injury [16]. The enzymatic antioxidant system includes superoxide dismutase (SOD), catalase (CAT), peroxidase (POD), and ascorbate peroxidase (APX) [17]. SOD catalyzes the dismutation of superoxide anion (O_2_^−^) into H_2_O_2_ and O_2_, CAT and POD further decompose H_2_O_2_ into H_2_O and O_2_ [18]. On the non-enzymatic side, metabolites such as ascorbic acid (ASA), glutathione (GSH), phenolic acids, and flavonoids serve as potent radical scavengers. In addition to their direct neutralizing activity, these compounds function as key metabolites in the ASA–GSH cycle, functioning as critical redox regulators [19].

Low-temperature storage is an effective strategy for maintaining postharvest fruit quality. It suppresses respiration, delays nutrient loss and softening, and enhances antioxidant capacity, thereby slowing senescence [20]. Tomatoes stored at 4 °C and 14 °C maintained better appearance and quality, with less softening and water loss [21]. Peaches stored at 1 °C showed enhanced antioxidant capacity and improved ROS scavenging, which helped maintain cellular redox balance [22]. Most commercial fig cultivars tolerate mild cold stress, and refrigeration at 0–1 °C with 90–95% RH is the conventional preservation strategy reported in existing literature [23]. Previous fig postharvest research frequently employs 0 °C or 4 °C as cold storage temperatures [24,25]. A total of 4 °C and 25 °C were selected to form two contrasting storage conditions, which produce differential oxidative status and softening characteristics in fruit and facilitate the exploration of the regulatory relationship between ROS accumulation and cell wall breakdown.

Little attention has been paid to the postharvest preservation and physiological regulation of Xinjiang-grown ‘Jin’Aofen’ figs; most existing fig research targets varieties cultivated in temperate humid regions. A total of 4 °C and 25 °C were set as two distinct treatments. Using ‘Jin’Aofen’ fig fruit as experimental samples, this work explored the regulatory impacts of storage temperature on postharvest quality, cell wall turnover and antioxidant capacity.

## 2. Materials and Methods

### 2.1. Fruit Materials and Storage Treatments

Fresh figs (*Ficus carica* L. cv. ‘Jin’Aofen’) were harvested from Alar City, Xinjiang Uygur Autonomous Region. Figs of similar size and uniform ripeness, with no visible pest damage or physical defects, were transported to the lab. They were randomly allotted to two temperature treatments (4 ± 1 °C or 25 ± 1 °C) with 90–95% relative humidity (RH). Eight plates were placed at each temperature, with 12 figs per tray, and additional figs were reserved for backup samples. Each treatment uses a fixed set of 12 figs, divided into three groups for the measurement of color, respiration rate and decay. For firmness and TSS measurements, a variable set of 12 fruits are split into three groups instead. Specimens were collected on days 0 through 7 during the storage period. After sampling, the fruits were sliced, snap-frozen in liquid nitrogen, and held at −80 °C.

### 2.2. Determination of Decay Incidence and Color Parameters

Fruits exhibiting visible fungal growth, water-soaked lesions, or tissue maceration were counted as decayed.

Fruit color was measured using a handheld colorimeter (Model CR-400, Konica Minolta, Tokyo, Japan). Color readings were taken from three spots around the equator of each fruit, recording L*, a*, and b* values.

### 2.3. Determination of Firmness, Respiration Rate, TSS and TA

Fruit firmness was measured at the equatorial region using a texture analyzer (Model TMS-PILOT, Ensoul Technology Co., Ltd., Beijing, China). The operating parameters were set as follows: probe dwell time, 2 s; load cell, 500 N; deformation, 20%; and test speed, 60 mm min^−1^. The mean value was used as the final result.

For respiration rate determination, 12 fruits were divided into three groups and placed in a sealed container connected to a gas analyzer for 30 min (Model JFQ-315OH, Beijing Junfang Institute of Physical and Chemical Technology, Beijing, China). Measurements were recorded every 10 min. Three biological replicates were performed, and the average value was calculated according to the method of Gao et al. [26].

Total soluble solid (TSS) was determined using a portable refractometer (Model AK002B, Ceyou Technology Co., Ltd., Shenzhen, China), and the results were expressed as %.

Titratable acidity (TA) was measured by potentiometric titration according to Jiang et al. [27]. The fig sample (5 g) was ground and diluted to 100 mL, after 30 min of standing and centrifugation. An amount of 20 mL aliquot of the supernatant was then brought to pH 8.2 by dropwise addition of 0.1 M NaOH, and the consumption volume was used to calculate TA, expressed as % malic acid equivalents.

### 2.4. Determination of Non-Enzymatic Antioxidant Contents

Total phenol content (TPC) was estimated using the protocol of Fan et al. [28]. Fig samples were extracted with 60% ethanol, and the extract was reacted with Folin–Ciocalteu reagent and Na_2_CO_3_. After dark incubation, absorbance was measured at 760 nm. Phenolic levels were derived from a gallic acid standard curve.

Total flavonoid content (TFC) in figs was determined colorimetrically based on a previously described procedure [29]. The extract was reacted sequentially with sodium nitrite, aluminum nitrate, and sodium hydroxide. After dark incubation, absorbance at 510 nm was recorded.

For ASA and GSH measurements, the method of Cao et al. [30] was used. For AsA determination, figs were mixed with trichloroacetic acid, and the supernatant was reacted with ethanol, phosphoric acid–ethanol, BP–ethanol, and FeCl_3_–ethanol solution. Absorbance readings at 534 nm were converted to AsA content (mg∙100 g^−1^) using a standard curve.

For GSH determination, figs were extracted with TCA containing EDTA-Na_2_. The supernatant was incubated with phosphate buffer and DTNB at room temperature. Absorbance was measured at 412 nm. Results were calculated as µmol∙g^−1^.

### 2.5. Determination of Antioxidant Enzyme Activities and PPO Activity

The activities of CAT and POD were determined using commercial assay kits (Suzhou Grace Biotechnology Co., Ltd., Suzhou, China). The results were calculated as U∙g^−1^.

For SOD, APX, GR, and PPO, the activity assays followed the procedures described in Cao et al. [30]. Fig powder was homogenized with the corresponding extraction buffer and centrifuged. Enzyme activities were then assayed using the corresponding reaction systems. Absorbance was recorded at 560 nm for SOD, 290 nm for APX, 340 nm for GR, and 420 nm for PPO. Results were calculated as U∙g^−1^.

### 2.6. Determination of ROS and MDA Contents

The contents of O_2_^−^, H_2_O_2_ and MDA were determined using commercial assay kits. Results were calculated as nmol∙g^−1^ (O_2_^−^ and MDA) and µmol∙g^−1^ (H_2_O_2_).

### 2.7. Determination of Cell Wall Polysaccharides

Cell wall polysaccharides followed the procedures described in Cao et al. [30]. Briefly, 0.05 g of fig was extracted three times with 95% ethanol in a boiling water. Soluble pectin was extracted by suspending the pellet in distilled water at 50 °C for 30 min. Protopectin was then released from the residue by boiling in 0.5 M H_2_SO_4_ for 1 h. Absorbance readings at 530 nm were converted to galacturonic acid equivalents (%).

Cellulose content was determined using a cellulose assay kit (Suzhou Grace Biotechnology Co., Ltd., Suzhou, China) Results were calculated as mg∙g^−1^.

### 2.8. Determination of Cell Wall Enzyme Activities

Activities of cell wall-degrading enzymes were determined as described by Cao et al. [30]. One gram of fig sample was sequentially extracted with pre-cooled 95% ethanol, 80% ethanol, and buffer containing 1.8 M NaCl, followed by centrifugation after each step. The final supernatant was collected as the crude enzyme extract.

For PG and PME, the reaction contained buffer, substrate, and enzyme. CEL used 1.5 mL sodium carboxymethyl cellulose solution and 0.5 mL of enzyme extract. After 1 h at 37 °C, DNS was added and boiled for 5 min. Cooled samples were read at 540 nm. Activities were determined against a glucose standard (mg∙h^−1^∙g^−1^).

### 2.9. Statistical Analysis

All measurements were performed in triplicate, and expressed as mean ± standard deviation (SD) (*n* = 3). Data were processed with Microsoft Excel 2019. All data were analyzed by two-way factorial ANOVA (FANOVA) via SPSS 27.0 to evaluate temperature, storage time main effects and their interaction. Duncan’s test (*p* < 0.05) was applied for mean separation. Data visualization was performed with GraphPad Prism 10 and Origin 2024.

## 3. Results

### 3.1. Effects of Storage Temperature on Appearance, Decay Incidence and Color Parameters

**Figure 1:**
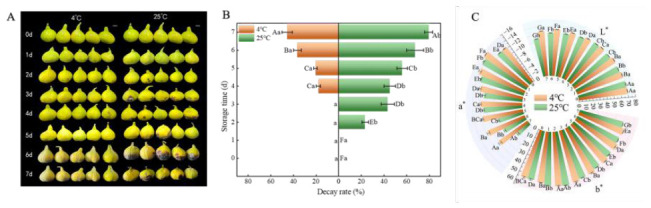
Effects of different storage temperatures on the appearance (**A**), decay incidence (**B**) and color parameters (**C**) of figs. Two-way FANOVA showed significant temperature × storage time interaction (*p* < 0.01). Uppercase letters denote differences across storage days under identical temperature; lowercase letters indicate temperature differences at the same sampling day (*p* < 0.05).

As storage progressed, figs gradually exhibited browning, softening, and decay symptoms (Figure 1A). Compared with stored at 25 °C, those at 4 °C maintained a better appearance, and no obvious browning or mold symptoms were observed during the first 5 days of storage. In contrast, figs stored at 25 °C showed faster peel darkening, yellowing, and visible decay.

Decay incidence is one of the most direct indicators for evaluating postharvest quality deterioration and shelf life of fruits [31]. No significant difference in decay incidence was observed at the early stage of storage, whereas the difference became significant thereafter (*p* < 0.05) (Figure 1B). By the end of storage, decay incidence reached about 80% in figs stored at 25 °C, compared with 45% in those stored at 4 °C.

Fruit color serves as a critical parameter for evaluating freshness and sensory quality [32]. The L* value gradually decreased (Figure 1C), but figs stored at 4 °C maintained higher L* values than those at 25 °C (*p* < 0.05). The a* value increased continuously in both groups, with higher values in figs stored at 25 °C than in those at 4 °C (*p* < 0.05). For both groups, the b* value peaked before declining. During the later stage of storage, figs stored at 4 °C showed higher b* values than those at 25 °C (*p* < 0.05).

### 3.2. Effects of Storage Temperature on Firmness, Respiration Rate, TSS, and TA

**Figure 2:**
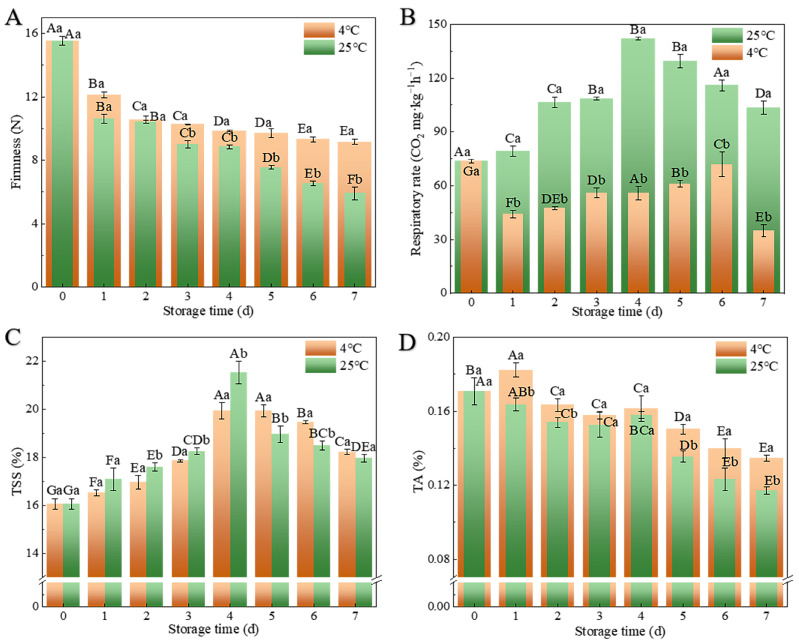
Effects of different storage temperatures on firmness (**A**), respiration rate (**B**), TSS (**C**) and TA (**D**) of figs. Two-way FANOVA showed significant temperature × storage time interaction (*p* < 0.01). Uppercase letters denote differences across storage days under identical temperature; lowercase letters indicate temperature differences at the same sampling day (*p* < 0.05).

Microbial infection during storage elevates decay rate and induces fruit softening, which is directly reflected by reduced firmness. During storage, fig firmness decreased continuously, with a sharp decline on day 1 (Figure 2A). Although no significant difference was observed in the early stage, figs at 4 °C maintained significantly higher firmness than at 25 °C from day 3 onward (*p* < 0.05). By day 7, firmness at 4 °C was 1.55-fold that at 25 °C.

Storage temperature significantly affected the respiration rate of figs (Figure 2B). Throughout the storage period, figs at 25 °C showed a significantly higher respiration rate than at 4 °C (*p* < 0.05). Storage at 4 °C delayed the respiratory peak by 2 days and reduced its maximum value by about 50%.

TSS first increased and then decreased, peaking on day 4 in both groups (Figure 2C). It was significantly lower at 4 °C in the early stage (*p* < 0.05), but declined more slowly thereafter. By the end of storage, figs stored at 4 °C retained higher TSS, which is consistent with findings reported by Zheng et al. [33]. TA content showed an overall decreasing trend in figs (Figure 2D). TA content was significantly better preserved at 4 °C than at 25 °C, with the greatest differences noted on day 1 and in the final phase of storage (*p* < 0.05). At the end of storage, the TA at 4 °C was 1.15-fold that at 25 °C.

### 3.3. Effects of Storage Temperature on Non-Enzymatic Antioxidants in Figs

**Figure 3:**
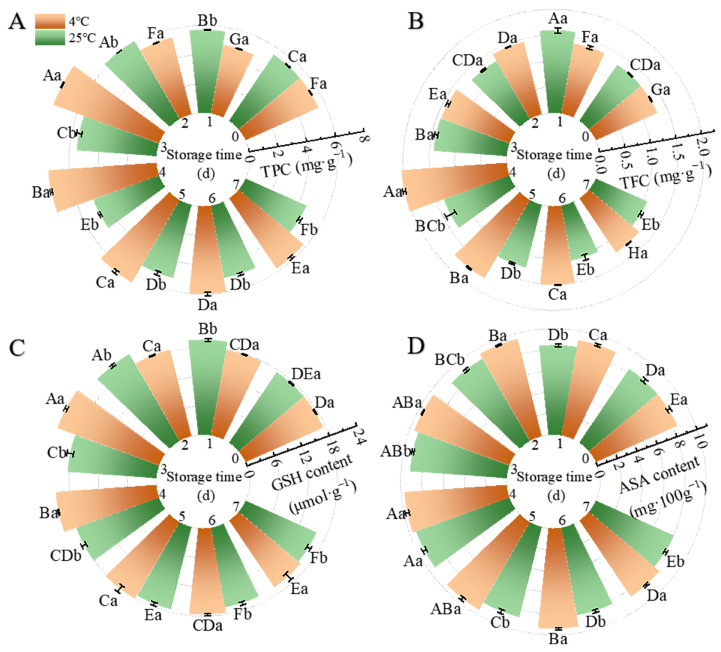
Effects of different storage temperatures on the TPC (**A**), TFC (**B**), GSH (**C**) and AsA (**D**) in figs. Two-way FANOVA showed significant temperature × storage time interaction (*p* < 0.01). Uppercase letters denote differences across storage days under identical temperature; lowercase letters indicate temperature differences at the same sampling day (*p* < 0.05).

TPC increased initially and then decreased (Figure 3A). It peaked on day 2 at 25 °C and on day 4 at 4 °C, with values of 6.18 and 7.42 mg·g^−1^, respectively. By day 7, TPC content had decreased to 5.82 mg·g^−1^ at 25 °C and 4.57 mg·g^−1^ at 4 °C. TFC showed a similar trend (Figure 3B). It reached a peak of 2.04 mg·g^−1^ on day 4 at 4 °C. During the later stage, TFC remained significantly higher at 4 °C than at 25 °C (*p* < 0.05).

GSH content also increased first and then decreased (Figure 3C). During the first 2 days, it was significantly lower at 4 °C than at 25 °C (*p* < 0.05). However, from day 3 onward, GSH content became higher at 4 °C. AsA content increased during the early stage (Figure 3D). From day 4 onward, it declined in both groups, but the decrease was more pronounced at 25 °C than at 4 °C. By day 7, AsA content remained at 8.69 mg·100 g^−1^ at 4 °C, compared with 8.06 mg·100 g^−1^ at 25 °C. The difference was significant throughout storage (*p* < 0.05).

### 3.4. Effects of Storage Temperature on Antioxidant Enzyme Activities and PPO Activity in Figs

**Figure 4:**
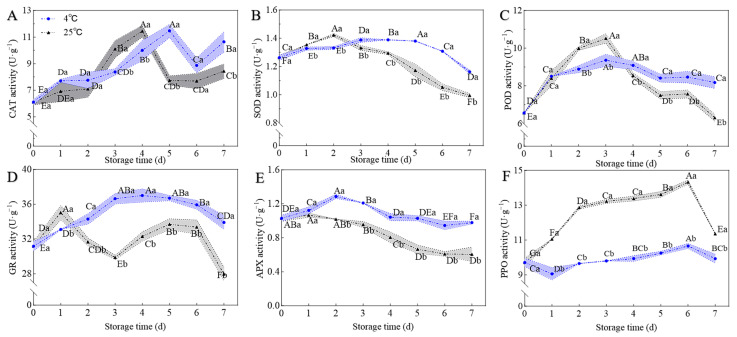
Effects of different storage temperatures on the activities of CAT (**A**), SOD (**B**), POD (**C**), GR (**D**), APX (**E**) and PPO (**F**) in figs. Two-way FANOVA showed significant temperature × storage time interaction (*p* < 0.01). Uppercase letters denote differences across storage days under identical temperature; lowercase letters indicate temperature differences at the same sampling day (*p* < 0.05).

CAT activity fluctuated throughout storage, rising then falling with a slight recovery (Figure 4A). It peaked on day 4 at 25 °C and on day 5 at 4 °C. From day 5 onward, CAT activity at 4 °C was generally higher than t at 25 °C, with significant differences observed on days 5 and 6 (*p* < 0.05). SOD activity increased initially and then declined (Figure 4B). At 25 °C, SOD activity peaked at 1.42 U g^−1^ on day 2. From day 3 onward, SOD activity at 4 °C was significantly higher than at 25 °C (*p* < 0.05). At the end of storage, figs stored at 4 °C still markedly higher SOD activity than those at 25 °C.

POD activity showed a trend similar to that of SOD (Figure 4C). In both groups, it peaked on day 3. The peak values at 4 °C and 25 °C were 9.36 and 10.51 U g^−1^, respectively. Thereafter, POD activity at 4 °C was significantly higher than at 25 °C (*p* < 0.05). By day 7, POD activity decreased to 8.18 U g^−1^ at 4 °C, whereas that in figs stored at 25 °C was only 77.14% of this value.

Except for days 0 and 1, GR activity was significantly higher at 4 °C than at 25 °C (*p* < 0.05) (Figure 4D). GR activity reached its peak on day 4 and then declined slightly at 4 °C. In contrast, GR activity remained at a relatively low level t 25 °C during most of the storage period and increased only in the later stage. By the end of storage, GR activity was still higher at 4 °C. The pattern of APX activity in figs stored at 25 °C differed markedly from that at 4 °C throughout storage (Figure 4E). It declined continuously from 1.03 to 0.61 U g^−1^ at 25 °C. By contrast, APX activity at 4 °C increased to 1.29 U g^−1^ on day 2 and then remained relatively stable. During the later stage, APX activity remained consistently higher at 4 °C.

PPO activity showed a bell-shaped trend in both groups (Figure 4F). At 25 °C, PPO activity increased rapidly and reached a peak of 14.33 U g^−1^ on day 6. Throughout storage, 4 °C storage markedly suppressed PPO activity relative to 25 °C (*p* < 0.05).

### 3.5. Effects of Storage Temperature on ROS and MDA in Figs

**Figure 5:**
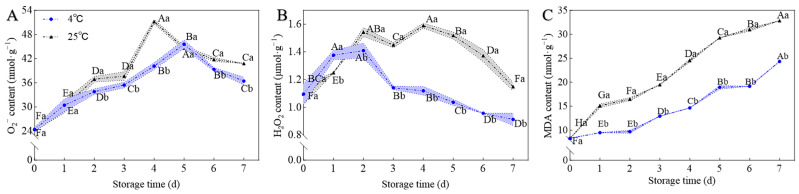
Effects of different storage temperatures on the contents of O_2_^−^ (**A**), H_2_O_2_ (**B**) and MDA (**C**) in figs. Two-way FANOVA showed significant temperature × storage time interaction (*p* < 0.01). Uppercase letters denote differences across storage days under identical temperature; lowercase letters indicate temperature differences at the same sampling day (*p* < 0.05).

ROS accumulation is another major factor contributing to postharvest deterioration in fruits [34].The contents of O_2_^−^ and H_2_O_2_ showed similar changing patterns during storage, both increasing initially and then decreasing (Figure 5A,B). At 25 °C, both O_2_^−^ and H_2_O_2_ contents reached their peak values on day 4. Compared with 25 °C, O_2_^−^ and H_2_O_2_ contents at 4 °C were 22% and 30% lower, respectively. On day 7, O_2_^−^ content was significantly lower at 4 °C than at 25 °C (*p* < 0.05), with values of 36.44 and 40.81 nmol·g^−1^, respectively. H_2_O_2_ content was significantly lower at 4 °C during the later stage (*p* < 0.05), and by day 7, it was reduced by 21% compared with that at 25 °C.

The MDA content increased continuously during storage (Figure 5C). Throughout the storage period, the MDA content at 25 °C was significantly higher than that at 4 °C (*p* < 0.05). By day 7, the MDA contents at 4 °C and 25 °C reached 24.30 and 38.24 nmol·g^−1^, respectively, representing increases of 2.0-fold and 3.8-fold compared with day 0.

### 3.6. Effects of Storage Temperature on Cell Wall Polysaccharides in Figs

**Figure 6:**
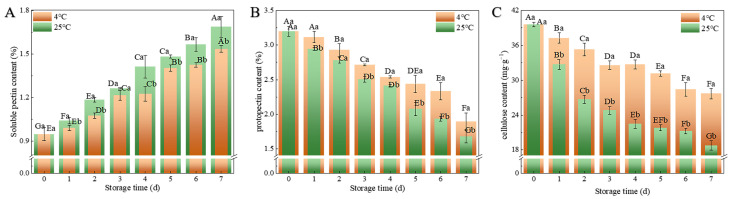
Effects of different storage temperatures on soluble pectin (**A**), protopectin (**B**) and cellulose (**C**) in figs. Two-way FANOVA showed significant temperature × storage time interaction (*p* < 0.01). Uppercase letters denote differences across storage days under identical temperature; lowercase letters indicate temperature differences at the same sampling day (*p* < 0.05).

The soluble pectin content gradually increased in both groups (Figure 6A). Except on days 0 and 3, it was lower at 4 °C than at 25 °C (*p* < 0.05). The greatest difference appeared on day 4, when the soluble pectin content at 4 °C was 13.23% lower than that at 25 °C. By day 7, the soluble pectin content at 25 °C was 1.10-fold that at 4 °C.

Protopectin content showed a gradual declining trend during storage (Figure 6B). Except for days 0 and 2, it remained significantly higher at 4 °C than at 25 °C (*p* < 0.05). The difference became more pronounced during the later stage. By day 7, the protopectin content at 4 °C was 1.13-fold that at 25 °C.

Cellulose content decreased sharply at 25 °C (Figure 6C). Compared with day 0, it decreased by 58.63% on day 7. In contrast, cellulose content at 4 °C declined more gradually and remained significantly higher throughout the storage period (*p* < 0.05). By day 7, the cellulose content at 4 °C was 1.48-fold that at 25 °C.

### 3.7. Effects of Storage Temperature on Cell Wall-Degrading Enzyme Activities in Figs

**Figure 7:**
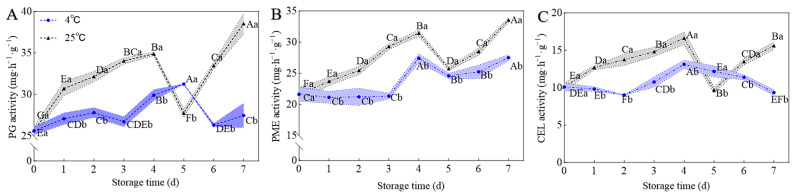
Effects of different storage temperatures on the activities of PG (**A**), PME (**B**) and CEL (**C**) in figs. Two-way FANOVA showed significant temperature × storage time interaction (*p* < 0.01). Uppercase letters denote differences across storage days under identical temperature; lowercase letters indicate temperature differences at the same sampling day (*p* < 0.05).

Cell wall degradation is recognized as a primary cause of postharvest softening in fig fruit [35], a process in which PG, PME, and CEL are key contributors [36]. Except on day 5, PG activity was significantly lower at 4 °C than at 25 °C (*p* < 0.05) (Figure 7A). By day 7, PG activity at 25 °C reached 38.52 mg·h^−1^·g^−1^, which was 1.4-fold than at 4 °C.

Throughout the storage period, PME activity at 25 °C was significantly higher than at 4 °C (Figure 7B). In both groups, PME activity reached a peak on day 4, with values of 31.42 and 27.40 mg·h^−1^·g^−1^ in figs stored at 25 and 4 °C, respectively. Although PME activity decreased in both groups on day 5, the difference between the two groups remained significant (*p* < 0.05).

CEL activity gradually increased from day 0 to day 4 in both groups (Figure 7C). Except on day 5, it was significantly lower at 4 °C than at 25 °C (*p* < 0.05). On day 4, CEL activity increased to 16.63 mg·h^−1^·g^−1^ at 25 °C and 13.12 mg·h^−1^·g^−1^ at 4 °C. During the later stage, CEL activity declined to 9.36 mg·h^−1^·g^−1^ at 4 °C, whereas that at 25 °C showed a slight increase.

### 3.8. Correlation Analysis

**Figure 8:**
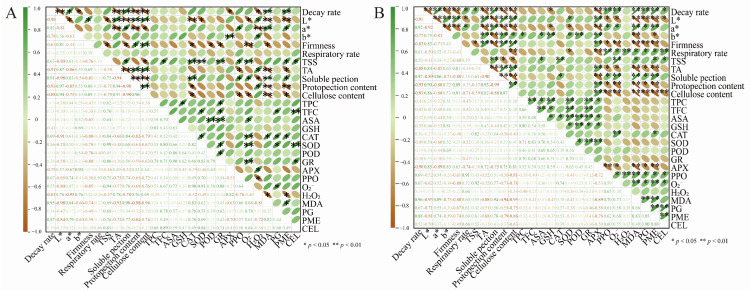
The correlation analysis heat map of the parameters of figs stored at 4 °C (**A**) and 25 °C (**B**).

Storage temperature markedly influenced the correlations among physiological parameters in figs. Under 4 °C storage (Figure 8A), firmness was positively linked to protopectin and cellulose, but negatively linked to soluble pectin (*p* < 0.05). PME exhibited a positive correlation with soluble pectin, while its relationship with protopectin and cellulose was negative (*p* < 0.05), indicating its involvement in cell wall degradation. Positive correlations were observed between the antioxidant enzymes and the non-enzymatic antioxidants. In particular, AsA showed extremely significant positive correlations with POD and GR (*p* < 0.01), suggesting coordinated action between enzymatic and non-enzymatic antioxidant systems. MDA was significantly correlated with L*, a*, and b* values, indicating a close relationship between membrane lipid peroxidation and peel browning.

Under 25 °C storage (Figure 8B), PME was positively correlated with PG and CEL (*p* < 0.01). In addition, PME correlated positively with soluble pectin and negatively with protopectin and cellulose (*p* < 0.05). Protopectin and cellulose were positively correlated with firmness. Soluble pectin was negatively correlated with firmness (*p* < 0.01). ROS were negatively correlated with protopectin and cellulose and positively correlated with soluble pectin, while cell wall-degrading enzymes were positively correlated with ROS, especially O_2_^−^. Respiration rate was significantly positively correlated with ROS and MDA (*p* < 0.05), and PPO was also positively correlated with ROS and MDA, suggesting that room-temperature storage promoted oxidative stress, membrane damage, and browning.

## 4. Discussion

Fresh figs have an extremely short postharvest shelf life. After ripening, softening, cracking, and ostiole enlargement accelerate pathogen invasion and juice leakage, ultimately resulting in rapid decay and deterioration [37]. Low-temperature storage is one of the most effective physical methods for delaying senescence and maintaining postharvest quality. Similar effects have been reported in pear [20], mango [38] and apple [39]. In the present study, storage at 4 °C delayed the respiratory climacteric by 2 days and maintained a lower respiration rate, because excessive respiration speeds up the consumption of TSS and TA [40]. This is consistent with the marked declines in fruit firmness, TSS, and TA observed under storage at 25 °C. Therefore, low-temperature storage can delay fig senescence by suppressing respiratory metabolism and reducing nutrient substrate consumption, which is in agreement with findings reported for pitaya and other horticultural crops [41].

Cell wall degradation acts as the core cause of postharvest fruit softening, and cell wall metabolism is closely linked to cellular redox status [42]. Excessive ROS disrupts redox homeostasis, aggravates membrane lipid peroxidation, damages membrane integrity, and accelerates fruit senescence. MDA, the end product of lipid peroxidation, is widely used as a key indicator of oxidative damage in plant tissues [43,44]. In this study, MDA, O_2_^−^, and H_2_O_2_ levels were higher at 25 °C than at 4 °C. This indicates more severe oxidative stress and membrane damage under ambient storage. In the present study, the activities of PME, PG, and CEL were all lower at 4 °C than at 25 °C. This further supports the inhibitory effect of low temperature on cell wall degradation. Cold treatment retained higher protopectin and cellulose alongside less soluble pectin, which retarded polysaccharide hydrolysis and structural degradation of cell walls. Compared with 25 °C, storage at 4 °C more effectively maintained protopectin and cellulose contents and partially inhibited protopectin hydrolysis.

CAT, SOD, and POD are key antioxidant enzymes involved in ROS scavenging [45]. The 4 °C treatment retained markedly higher activities of these three enzymes relative to 25 °C, which facilitated the elimination of excess ROS. Consistent with prior postharvest studies, enhanced antioxidant enzyme activity alleviates ROS overaccumulation and lipid peroxidation to sustain membrane integrity [46,47]. The AsA–GSH cycle is a central antioxidant pathway [48]. In this cycle, APX reduces H_2_O_2_ by utilizing AsA as an electron donor, while GSH is regenerated through the action of GR, thereby continuously replenishing antioxidant substrates and sustaining intracellular redox balance [49,50]. In the present study, APX and GR activities were significantly higher at 4 °C than at 25 °C, especially during the later stage. Meanwhile, 4 °C storage maintained relatively high AsA levels and preserved GSH more effectively in late storage. These results indicate that low-temperature storage not only enhances the enzymatic scavenging capacity, but also supports the regeneration and accumulation of antioxidant substances. Similar cold-induced improvement of the AsA–GSH cycle has been documented in broccoli and pineapple [51,52]. As vital secondary metabolites, phenolics and flavonoids participate in fruit ripening and stress responses and act as core components of the non-enzymatic antioxidant system [53,54]. In the later storage period, TPC and TFC declined gradually, but both remained significantly higher at 4 °C than at 25 °C. These indicate that storage at 4 °C effectively retards the depletion of antioxidant compounds throughout senescence. These polyphenolic compounds directly neutralize ROS and coordinate with the AsA–GSH cycle and antioxidant enzymes to stabilize cellular redox status. Accordingly, conserving both enzymatic and non-enzymatic antioxidants strengthens the fruit’s overall antioxidant defense network under cold preservation.

Browning is one of the most visible symptoms of postharvest senescence in figs. PPO and POD are the primary enzymes responsible for enzymatic browning. They catalyze phenolic oxidation, which leads to peel discoloration [55]. PPO is a key rate-limiting enzyme in enzymatic browning, as it oxidizes phenolics into quinones that subsequently form brown pigments [56]. Although PPO activity showed an initial increase followed by a decline, it remained significantly higher at 25 °C than at 4 °C. This indicates that low-temperature storage effectively suppresses enzymatic browning in figs. This finding aligns with the color variations recorded throughout storage. Visually, figs stored at 25 °C showed a clear transition in peel color from green to yellow, whereas figs stored at 4 °C maintained higher L* and b* values and lower a* values, indicating less browning and slower yellowing.

**Figure 9:**
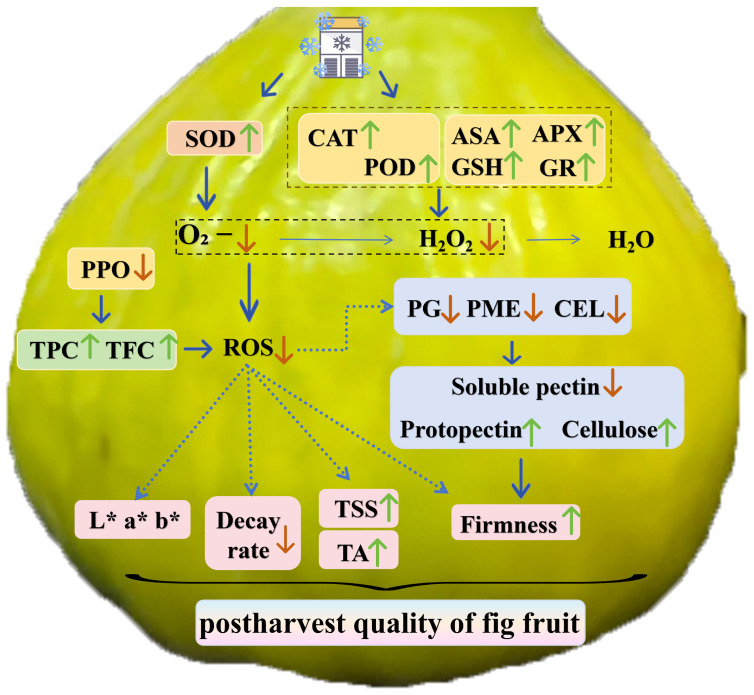
Mechanism of maintaining fig quality at low temperature.

A comprehensive regulatory model illustrating the crosstalk between redox homeostasis, antioxidant system and cell wall metabolism in postharvest fig softening is summarized in Figure 9. Redox homeostasis is a key link between antioxidant systems and cell wall metabolism. It functions as an important modulator of postharvest fruit softening. Physiological data and correlation analysis showed that ‘Jin’Aofen’ figs stored at ambient temperature had excessive ROS accumulation and severe membrane lipid peroxidation. These changes were significantly positively correlated with high activities of cell wall-degrading enzymes and rapid degradation of cell wall polysaccharides. Excessive ROS accelerates fruit softening in two ways. It damages the structure of pectin and cellulose. It also activates cell wall-degrading enzymes. In mature fruits, accumulated H_2_O_2_ and ·OH degrade cell wall polysaccharides via non-enzymatic oxidation [57,58]. Exogenous H_2_O_2_ has been proven to upregulate cell wall-degrading enzymes in longan [15] and kiwifruit [59]. Stabilizing redox homeostasis can reverse such activation and slow cell wall degradation [60]. Low-temperature storage reduced ROS accumulation and oxidative damage, and maintained intracellular redox balance. Accordingly, low temperature may contribute to suppressing cell wall degradation through modulation of antioxidant metabolism. This consequently delays the degradation of cell wall polysaccharides and fruit softening. The significant correlations observed among ROS levels, oxidative injury and cell wall metabolism only provide supportive evidence for the regulatory mechanism proposed herein, but cannot confirm definitive causal relationships between these physiological indicators. Further targeted functional validation experiments are required to verify the direct causal interactions underlying fig fruit softening during storage.

## 5. Conclusions

This study shows that cold storage at 4 °C effectively preserves the postharvest quality of ‘Jin’Aofen’ figs. Low temperature enhances the activities of antioxidant enzymes. It also elevates the contents of non-enzymatic antioxidants. These changes reduce ROS accumulation and membrane lipid peroxidation. They help maintain cellular redox homeostasis. Meanwhile, cold storage suppresses the activities of cell wall-degrading enzymes. It delays the degradation of pectin and cellulose. This slows fruit softening. Overall, low temperature regulates antioxidant metabolism and maintains redox balance. It further inhibits cell wall degradation. Together, these effects synergistically delay postharvest senescence of fig fruit. The findings of this study can provide theoretical support and practical references for subsequent research on fig preservation, and facilitate the further development of refined postharvest preservation technologies for figs.

## Data Availability

The original contributions presented in the study are included in the article, further inquiries can be directed to the corresponding author.
